# The Role of Atrial Fibrillation and Oral Anticoagulation Status in Health‐Related Quality of Life 12 Months After Ischemic Stroke or TIA

**DOI:** 10.1002/brb3.70248

**Published:** 2025-01-08

**Authors:** Manuel C. Olma, Lena Steindorf‐Sabath, Serdar Tütüncü, Claudia Kunze, Cornelia Fiessler, Paulus Kirchhof, Joanna Dietzel, Johannes Schurig, Patrick Oschmann, Ludwig Niehaus, Christian Urbanek, Götz Thomalla, Darius G. Nabavi, Joachim Röther, Ulrich Laufs, Roland Veltkamp, Peter U. Heuschmann, Karl Georg Haeusler, Matthias Endres, for the MonDAFIS Investigators

**Affiliations:** ^1^ Center for Stroke Research Berlin Charité—Universitätsmedizin Berlin Berlin Germany; ^2^ Institute of Clinical Epidemiology and Biometry Julius‐Maximilians‐Universität Würzburg Würzburg Germany; ^3^ Institute of Cardiovascular Sciences University of Birmingham Birmingham UK; ^4^ Department of Cardiology University Heart and Vascular Center Hamburg Hamburg Germany; ^5^ German Center for Cardiovascular Research Hamburg Germany; ^6^ Institute of Social Medicine, Epidemiology and Health Economics Charité—Universitätsmedizin Berlin Berlin Germany; ^7^ Department of Neurology Hospital Bayreuth Bayreuth Germany; ^8^ Department of Neurology Rems‐Murr‐Hospital Winnenden Winnenden Germany; ^9^ Department of Neurology Clinical Center of Ludwigshafen Ludwigshafen Germany; ^10^ Department of Neurology University Medical Center Hamburg‐Eppendorf Hamburg Germany; ^11^ Department of Neurology Vivantes Hospital Neukölln Berlin Germany; ^12^ Department of Neurology Asklepios Hospital Altona Hamburg Germany; ^13^ Department of Cardiology University Hospital Leipzig University Leipzig Germany; ^14^ Department of Neurology Alfried Krupp Krankenhaus Essen Germany; ^15^ Department of Brain Sciences Imperial College London London UK; ^16^ Clinical Trial Center Würzburg University Hospital Würzburg Würzburg Germany; ^17^ Department of Neurology University Hospital Ulm Ulm Germany; ^18^ German Center for Neurodegenerative Diseases (DZNE) Partner Site Berlin Berlin Germany; ^19^ German Center for Cardiovascular Research (DZHK) Partner Site Berlin Berlin Germany; ^20^ Excellence Cluster NeuroCure Berlin Germany; ^21^ German Center for Mental Health (DZPG), Partner Site Berlin Berlin Germany; ^22^ Department of Neurology with Experimental Neurology Charité—Universitätsmedizin Berlin Berlin Germany

**Keywords:** atrial fibrillation, ischemic stroke, oral anticoagulation, quality of life, transient ischemic attack

## Abstract

**Aims:**

Atrial fibrillation (AF) accounts for about 20% of all ischemic strokes worldwide. It is known that AF impairs health‐related quality of life (HRQOL) in the general population, but data on HRQOL in stroke patients with newly diagnosed AF are sparse.

**Methods:**

Post hoc analysis of the prospective, investigator‐initiated, multicenter MonDAFIS study (NCT02204267) to analyze whether AF‐related oral anticoagulation (OAC), and/or AF‐symptom severity are associated with HRQOL after ischemic stroke or transient ischemic attack (TIA). HRQOL was measured using the EQ‐5D‐3L‐questionnaire (including EQ‐index/EQ‐VAS) at baseline and after 12 months using multivariable linear mixed models. AF symptom severity was assessed using the European Heart Rhythm Association classification and symptom severity score (EHRA score) categorizing patients with no/mild/severe/disabling AF‐related symptoms.

**Results:**

A first episode of AF was detected in 261/2927 (8.9%) patients within 12 months after the index stroke and 227/2920 (7.8%) patients had AF and were anticoagulated at 12 months. HRQOL (measured by EQ‐index, *n* = 2495 patients) was higher in AF patients on OAC compared to AF patients without OAC at 12 months after stroke (mean difference: MD: –16.8, 95% CI: 5.6 to 28.0), and similar in AF patients under OAC compared with patients without AF (MD: 2.0, 95% CI: –2.2 to 6.3). AF‐related symptoms were negatively associated with HRQOL (measured by EQ‐index) indicating that stroke patients with AF‐related symptoms had a lower HRQOL compared to asymptomatic AF patients (mild vs. asymptomatic: MD: –9.0, 95% CI: –17.7 to –0.3; severe/disabling vs. asymptomatic: MD: –19.1, 95% CI: –34.7 to –3.4).

**Discussion:**

Stroke patients with newly diagnosed AF are at risk of lower quality of life at 12 months, depending on OAC status and AF symptom severity.

## Graphical abstract

This post‐hoc analysis of the prospective MonDAFIS study (NCT02204267) analyzed whether oral anticoagulation (OAC) and symptom severity of atrial fibrillation (AF) are associated with health‐related quality of life (HRQOL) in stroke patients with newly diagnosed AF. HRQOL was examined using EQ‐index at baseline and 12 months in 2,540 patients. Patients with AF on OAC showed higher HRQOL (EQ‐index) at 12 months compared to AF‐patients without OAC, while greater AF‐symptom severity correlated with lower HRQOL (EQ‐index) at 12 months post‐stroke.

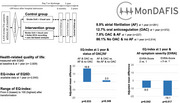

## 1 Introduction

Cardioembolic stroke accounts for approximately 20–30% of ischemic strokes worldwide, is mostly due to atrial fibrillation (AF), and is associated with a high morbidity and mortality (Findler et al. 2017; Hewage et al. 2023).

Stroke‐related neurological deficits affect activities of daily living, social well‐being, and heath related quality of life (HRQOL) (Katzan et al. 2018; Green and King 2010; Yeoh et al. 2018). However, longitudinal data on HRQOL in stroke patients with newly diagnosed AF are sparse (Sadlonova et al. 2021). There is evidence from a registry‐based study and a prospective cohort study (conducted in the United States) that patients with minor stroke also have a lower HRQOL compared to stroke‐free individuals (Lai et al. 2002; Sangha et al. 2015). A systematic review that identified five observational studies conducted in Europe or North America reported that patients with AF have poorer HRQOL than healthy controls or than unselected patients of the general population (Thrall et al. 2006), and AF‐related symptoms are strongly associated with reduced HRQOL (Wynn et al. 2014).

Vitamin K antagonists (VKAs) have been a longstanding standard for preventing thromboembolic events in stroke patients with AF. Within the last decade, nonvitamin K–dependent oral anticoagulants (NOACs) have emerged as an alternative treatment (Liu et al. 2020). While evidence‐based medicine has traditionally guided treatment strategies, patient‐reported outcomes are increasingly recognized as crucial measures in clinical trials. Limitations of the treatment with VKA are a narrow therapeutic range requiring regular monitoring of international normalized ratio (INR), multiple drug interactions and dietary restriction, all of which are not required in the treatment with NOAC (Gomez‐Outes et al. 2013). It is therefore conceivable that the results reported by patients are influenced by these factors. Whether or not the use of NOAC and VKA impacts on patient reported outcome measurements (such as specific HRQOL questionnaires, satisfaction, adherence or symptom burden) was analyzed in a systematic review (Liu et al. 2020). With regard to HRQOL, this question is still to be debated as no statistical inference could be performed in this systematic review due to heterogeneity of the included studies (one randomized controlled study and five observational studies) (Afzal et al. 2019).

In summary, current knowledge about how newly diagnosed AF affects HRQOL in stroke patients is limited and inconclusive. Therefore, increasing this knowledge is crucial for developing patient‐centered treatment strategies. This post hoc analysis of the prospective, multicenter MonDAFIS study addresses this need by examining the association of HRQOL and AF, which was newly diagnosed within 12 months after ischemic stroke or TIA, while differentiating symptom severity of AF and anticoagulation status.

## 2 Methods

### 2.1 Study Design and Patient Population

The MonDAFIS study was an investigator‐initiated, prospective, randomized, multicenter study, sponsored by the Charité—Universitätsmedizin Berlin, Germany and supported by an unrestricted research grant from Bayer Vital GmbH, Germany to the Charité. The Bayer Vital GmbH Germany had no influence on study design, study protocol, collection, analysis, and interpretation of data, writing and submitting the paper for publication. The MonDAFIS study was approved by the ethics committees of all participating sites, first by the Charité Ethics Committee, Berlin, Germany (EA2_033_14). All study patients gave written informed consent. This trial was registered with ClinicalTrials.gov, NCT02204267. Study patients without known AF were randomized 1:1 to continuous Holter‐ECG recording for up to 7 days during the in‐hospital stay of index stroke or TIA on top of standard of diagnostic care (intervention group) or to standard diagnostic care (control group) for AF detection in an open label fashion. The primary outcome of the MonDAFIS study was the proportion of patients on oral anticoagulants (NOAC or VKA) at 12 months after the index event. The study rationale and design as well as the main results were both published previously, along with the study protocol and the statistical analysis plan (Gomez‐Outes et al. 2013; Afzal et al. 2019). The aim of this secondary analysis was i) to assess the impact of newly detected AF and its symptom severity on HRQOL in stroke patients, ii) whether the anticoagulation status, and, iii) the type of oral anticoagulation (NOAC vs. VKA), differentially affect HRQOL during follow‐up. AF symptom severity was characterized using the European Heart Rhythm Association classification and symptom severity score (EHRA score), which differentiates between 1 point (no symptoms), 2 points (mild symptoms, normal daily activities not affected), 3 points (severe symptoms, normal daily activities affected), and 4 points (disabling symptoms, discontinued normal daily activities) (Hindricks et al. 2021).

### 2.2 Outcome Measures: Health‐Related Quality of Life

In this post hoc analysis of the MonDAFIS study, clinical data and outcomes of patients were analyzed with regard to HRQOL (Haeusler et al., 2021). HRQOL was measured using the validated EQ‐5D‐3L, consisting of two parts: the EQ‐5D descriptive system and the EQ‐5D visual analogue scale (EQ‐VAS). The EQ‐5D‐3L descriptive system comprises five dimensions of different aspects of health: mobility, self‐care, usual activities, pain/discomfort, and anxiety/depression. Each of the five dimensions has three response levels (“no problems,” “some problems,” and “extreme problems”) representing the health states. These health states can be converted into a single value (EQ‐index) by using valuations of health states that have been generated for different populations (Dolan, 1997). In the MonDAFIS study, we used the valuation set based on a representative population from Germany (Greiner et al. 2005). Health state index scores generally range from less than –1 to 1 where 0 represents a state equivalent to death, 1 represents perfect health and negative values represent states that are considered worse than death. In contrast, the EQ‐VAS records the patient's own assessment of their health status. Furthermore, the EQ‐VAS scores range from 100 to 0 (with 100 being the best imaginable health and 0 worst imaginable health). In this study, HRQOL was measured at baseline and at 12 months follow‐up.

### 2.3 Statistical Analysis

Baseline characteristics are reported as frequencies and percentages for categorical variables or median and interquartile range (IQR) or means and standard deviation (SD) for metric variables. We used exact Fisher Test, Mann–Whitney *U* test, Kruskal–Wallis or *t*‐test for independent samples when appropriate regarding differences in univariate comparisons. Statistical analysis of AF on HRQOL was performed in the intention‐to‐treat population with available information for EQ‐index and EQ‐VAS at 12 months after the index event. The EQ5D‐questionnaire addressed in its last question whether or not the questionnaire could be answered unreservedly by the patients themselves (i.e., without the help of a proxy). If this was not confirmed, ED5D data was discarded.

The effect of newly detected AF on EQ‐index and EQ‐VAS was estimated using multivariable linear mixed models (main model) with no variable selection. We included AF, OAC (yes/no) at 12 months after the index event and their interaction in the main model. To simplify the interpretation of the interaction effect, estimated marginal means with 95% CI and marginal effects were calculated. Additional independent variables, for which the main models were adjusted, are listed in the supplement.

We performed two subgroup analyses: subgroup model 1 included the same independent variables as the main model but was restricted to stroke/TIA patients with detected AF within 12 months. In order to analyze symptom severity related to AF, the EHRA score was additionally included (EHRA score 1 point, EHRA score 2 points, or EHRA score ≥ 3 points). Subgroup model 2 was analogous to model 1 but AF symptom severity was replaced by the use and type of OAC (no OAC, NOAC or VKA). As residuals of the EQ‐index were not normally distributed, the EQ‐index value was scaled to values between 0 and 1 and then transformed by the following formula: (EQ‐index × 100)^4^/1,000,000. After transformation EQ‐index and EQ‐VAS ranged from 0 to 100. It is conceivable that missing data was not at random (MNAR), therefore no imputation procedures were carried out and a complete case analysis was conducted. All statistical analysis were done using R (version 4.00). Even though the main‐analyses of HRQOL was preplanned, no multiplicity adjustments were done for the two subgroup analysis. Thus, the results of these subgroup analyses have to be considered exploratory. *p* Values < 0.05 were considered statistically significant.

## 3 Results

### 3.1 Patient Characteristics

**FIGURE 1:**
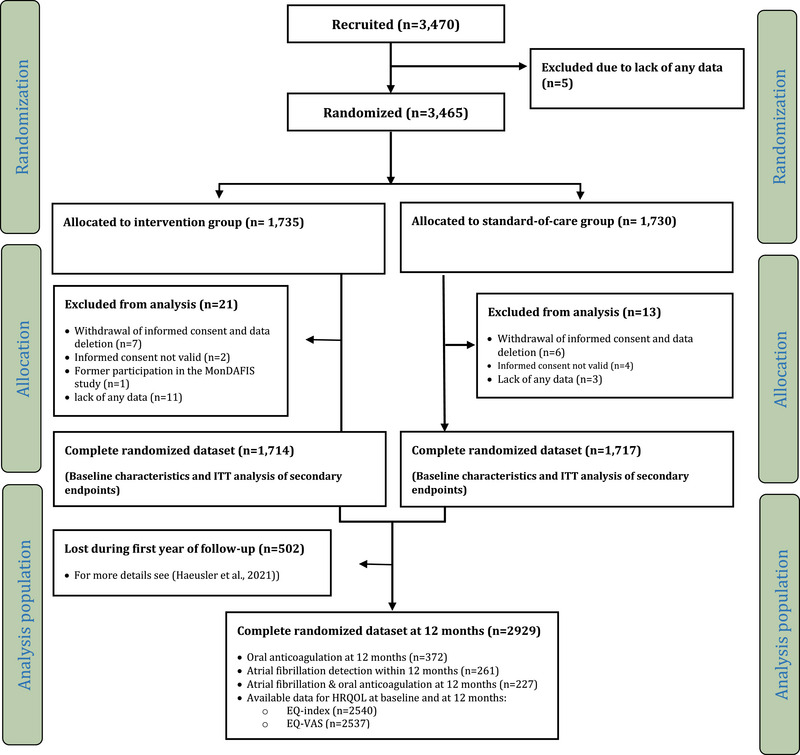
Trial profile of the secondary analysis of the MonDAFIS cohort; see also Haeusler et al. (2021).

A total of 3431 patients were analyzed. Mean age was 66.2 years (SD ± 12.9 years), 1356 patients (39.5%) were female and 1030 patients (30.1%) had a TIA (vs. stroke) as qualifying event (Figure 1). The median NIHSS scale score on admission was 2 points (Table S1). Overall, 3082/3431 patients (89.8%) had data on EQ‐5D available at baseline. Compared to patients with available EQ‐5D data at baseline, patients without EQ‐5D were slightly older, more frequently male, had more often severe strokes (i.e., median NIHSS score/ mRS ≥ 3), and more often underwent endovascular treatment or intravenous thrombolysis because of the index stroke (Table S1). Overall, follow‐up data at 12 months was available in 2929/3431 (85.4%) patients. Accordingly, 502 (14.6%) patients were lost during the first year of follow‐up due to death, withdrawal of informed consent, protocol violations, or lost‐to‐follow‐up (for more details, see Haeusler et al. 2021). Data on AF status at 12 months after the index stroke was available in 2927 (99.9%) patients. AF was newly diagnosed in 261/2927 patients (8.9%) within 12 months after stroke/TIA (intervention group 144/1486 (9.7%) patients; control group: 117/1441 (8.1%) patients). Compared to patients without AF, patients with AF were older, more often female, had a lower level of education, had more cardiovascular risk‐factors, and received intravenous thrombolysis treatment more often (Table S2). Overall, 227/2920 (7.8%) patients with newly detected AF were on OAC at 12 months follow‐up. Of the 261 patients with newly diagnosed AF within 12 months, 26/261 (10.0%) patients were on VKA and 201/261 (77.0%) on NOAC. Of note, patients with AF who did not receive OAC at 12 months had overall similar baseline characteristics compared to patients with AF with OAC (Table S3). Patients with AF but without OAC at 12 months differed only in few factors that are not linked to higher bleeding risk compared to patients with AF treated with OAC at 12 months: they had less often received intravenous thrombolysis, less frequent a body mass index ≥ 30 and the lowest proportion of patients with a score of ≥ 3 of the modified Rankin Scale (mRS).

### 3.2 Health‐Related Quality of Life and AF

**FIGURE 2:**
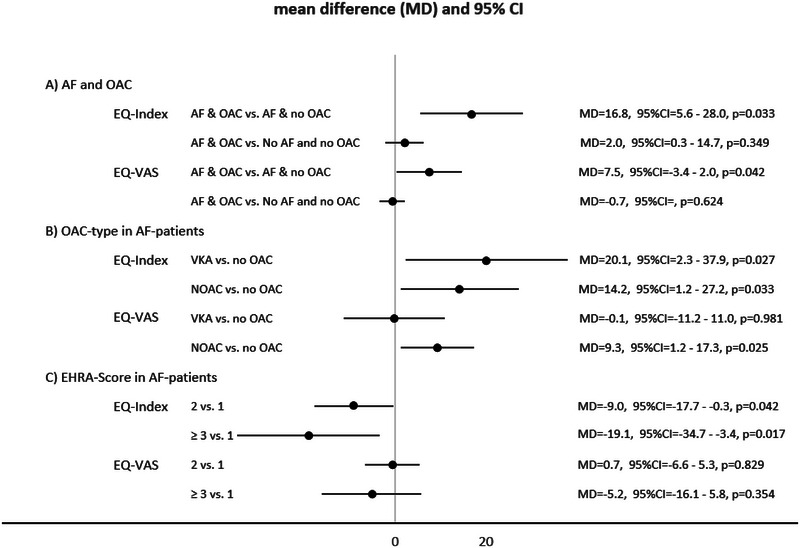
Forest plot of the mean differences of the multivariable linear regressions of Tables 2, 3, and S6 and corresponding 95% confidence intervals on EQ‐Index and EQ‐VAS at 12 months (variables for multivariable adjustments are listed in Tables 2, 3, and S6). (A) Estimated marginal means are depicted for the interaction AF × OAC (EQ‐Index/EQ‐VAS: *n* = 2495/2492), (B) subtype of OAC in patients in patients with AF only (EQ‐Index/EQ‐VAS: *n* = 195/194), and (C) AF‐symptom severity in patients with AF only stratified according to the EHRA score (EQ‐Index/EQ‐VAS: *n* = 218/217). *p* Values < 0.05 were considered statistically significant.

Overall, mean EQ‐index at 12 months follow‐up (70.0) was lower compared to the mean EQ‐index at baseline (76.0). Individual EQ‐5D levels at baseline and at 12 months follow‐up are listed in Tables S4a and S4b according to the type of index event. Mean EQ‐VAS at 12 months (70.9) was similar compared to mean EQ‐VAS at baseline (71.4). Univariate paired comparisons of EQ‐5D levels at baseline and at 12 months are listed in Table 1. Multivariable analysis of patients with AF (subgroup model 1, Table S6, Figure 2) demonstrated that AF‐related symptom severity (measured by the EHRA score) was negatively associated with EQ‐ index at 12 months. Mild versus no AF symptoms EQ‐index (mean difference = –9.04, *p* = 0.042; Table S6) as well as severe or disabling AF symptoms versus no AF symptoms (mean difference = –19.06, *p* = 0.017; Table S6) were statistically significantly associated with lower EQ‐index. The EQ‐VAS did not show an association with the EHRA score in patients with AF (*p* > 0.05, Table 3, Figure 2).

### 3.3 Association of OAC With Health‐Related Quality of Life in Patients With AF

There was a statistically significant interaction of OAC status and AF (EQ‐index: mean difference = –22.57, *p* (for interaction) < 0.001 and EQ‐VAS, mean difference = –13.71, *p* = 0.001) for a lower EQ‐index at 12 months after the index event (Table 2). Patients with AF but without OAC had a marginally lower HRQOL compared to patients with AF with OAC (EQ‐index: mean difference = –16.80, *p* = 0.033 and EQ‐VAS, mean difference = –7.47, *p* = 0.042). Patients with AF and OAC showed a similar HRQOL compared to patients without AF who were not anticoagulated (EQ‐index: mean difference = 2.02, *p* = 0.349; EQ‐VAS: mean difference = –0.68, *p* = 0.624). Multivariable subgroup‐analysis including type of OAC in patients with AF showed lower EQ‐index for non‐anticoagulated patients with AF compared to both OAC groups (NOAC and VKA) but no difference between NOAC and VKA at 12 months (subgroup model 2, Table 3, Figure 2). In contrast, EQ‐VAS at 12 months was lower in non‐anticoagulated patients with AF compared to NOAC‐treated patients with AF (mean difference = –9.38, *p* = 0.030). VKA‐treated patients had a similar EQ‐VAS at 12 months when compared to non‐anticoagulated patients (mean difference = 0.14, *p* = 0.981). Furthermore, the multivariable linear mixed model indicated that non‐anticoagulated patients with AF had a lower EQ‐index (mean difference = –14.78, *p* = 0.006) and EQ‐VAS at 12 months (mean difference = –8.15, *p* = 0.018; Table 2, Figure 2) when compared to patients without AF.

**TABLE 1:** Quality of life: EQ‐index and EQ‐VAS at baseline and 12 months after index stroke/TIA.

	Transformed EQ‐index					EQ‐VAS			
	Available data (*n*)	Available data on EQ‐index (*n*)	Mean	SE	*p* Value*	Available data on EQ‐VAS (*n*)	Mean	SE	*p* Value*
At baseline	3431	3095	73.22	0.59	< 0.001	3087	70.05	0.63	*p* = 0.332
At 12 months after the index event	2929	2747	69.86	0.62		2747	70.86	0.38	

*t‐test for dependent samples (at baseline vs. at 12 months) for the EQ‐Index (*n* = 2540) and for the EQ‐VAS (*n* = 2537). Both the transformed EQ‐index and the EQ‐VAS can range from 0 to 100.

**TABLE 2:** Quality of life: EQ‐index and EQ‐VAS 12 months after index stroke/TIA (analyzed by a multivariable linear mixed model*).


	EQ‐index at 12 months after the index event				EQ‐VAS at 12 months after the index event			
Fixed effects	Mean difference	SE	*p*	*R* ^2^ (in %)	Mean difference	SE	*p*	*R* ^2^ (in %)
(Intercept)	61.22	4.90			67.72	3.29		
EQ‐index at baseline	0.41	0.02	< 0.001	14.6	0.28	0.02	< 0.001	7.6
Age	−0.21	0.06	< 0.001	0.6	−0.15	0.04	< 0.001	0.7
Female sex	−5.86	1.20	< 0.001	0.9	−1.76	0.77	0.022	0.2
Education in years								
< 9								
9–10	1.28	2.00	0.522	0.0	1.23	1.27	0.335	0.0
≥ 11	4.26	1.45	0.003	0.3	2.21	0.93	0.018	0.2
NIHSS score on admission								
0 points								
1–4	−2.75	1.85	0.137	0.1	−1.05	1.19	0.375	0.0
≥ 5	−5.50	2.49	0.027	0.2	−3.94	1.59	0.013	0.2
Modified Rankin scale score on admission ≥ 3	−3.72	1.47	0.011	0.3	−3.43	0.93	< 0.001	0.5
Index stroke								
TIA								
Ischemic stroke	3.80	1.31	0.004	0.3	0.65	0.84	0.438	0.0
Endovascular treatment	−3.52	3.82	0.357	0.0	−2.49	2.45	0.309	0.0
Intravenous thrombolysis	4.04	1.55	0.009	0.3	4.28	0.99	< 0.001	0.7
Comorbidities								
Congestive heart failure	−5.44	3.75	0.146	0.1	−3.77	2.40	0.116	0.1
Chronic obstructive pulmonary disease	−6.78	3.04	0.026	0.2	−7.19	1.94	< 0.001	0.5
Hypertension	−3.05	1.49	0.041	0.2	−2.94	0.95	0.002	0.4
Diabetes mellitus	−2.44	1.41	0.082	0.1	−1.27	0.90	0.160	0.1
Hypercholesterolemia	0.00	1.19	0.998	0.0	−0.76	0.75	0.311	0.0
Current smoker	−2.78	1.22	0.023	0.2	−1.36	0.78	0.083	0.1
Prior vascular event	−4.98	1.40	< 0.001	0.5	−2.56	0.90	0.004	0.3
Arterial disease**	−1.66	1.78	0.351	0.0	−1.92	1.14	0.094	0.1
Body mass index ≥ 30 kg/m^2^	−3.58	1.36	0.008	0.3	−0.84	0.87	0.338	0.0
Renal impairment	−4.03	2.27	0.076	0.1	−3.21	1.45	0.027	0.2
SAE within 1 year after the index‐stroke								
TIA	−5.99	4.34	0.167	0.1	−6.14	2.78	0.027	0.2
Stroke	−5.00	2.82	0.076	0.1	−4.18	1.83	0.022	0.2
Major bleeding	−5.77	8.56	0.500	0.0	−15.18	5.50	0.006	0.3
Myocardial infarction	−4.07	6.24	0.514	0.0	−6.13	4.00	0.126	0.1
Atrial fibrillation	−14.78	5.38	0.006	0.3	−8.15	3.45	0.018	0.2
Oral anticoagulation at 12 months after the index stroke								
No oral anticoagulation								
Oral anticoagulation	−5.76	2.64	0.029	0.2	−6.25	1.70	< 0.001	0.5
Atrial fibrillation × oral anticoagulation								
Atrial fibrillation × no anticoagulation								
Atrial fibrillation × anticoagulation	22.57	6.28	< 0.001	0.5	13.71	4.03	0.001	0.5
								
Random effects
Intraclass correlation coefficient (ICC)	0.01				0.01			
Centers	38				38			
*n*	2495				2492			
Marginal *R* ^2^/conditional *R* ^2^	0.270/0.280				0.210/0.214			

Abbreviations: NIHSS, National Institute of Health Scale, EQ‐5D = EuroQOL–5 Dimension instrument for measuring quality of life, TIA = transient ischemic attack. SAE = serious adverse events.

*Analogue univariate linear regressions are listed in Table S5.

**Arterial disease comprises coronary heart disease and peripheral arterial disease.

**TABLE 3:** Subgroup analysis model 2: quality of life/EQ‐index and EQ‐VAS at 12 months in patients with atrial fibrillation diagnosed within 12 months after the index stroke/TIA stratified for treatment with oral anticoagulation analyzed by a multivariable linear mixed model.*

	EQ‐index at 12 months after the index event				EQ‐VAS at 12 months after the index event			
Fixed effects	Mean difference	SE	*p*	*R* ^2^ (in %)	Mean difference	SE	*p*	*R* ^2^ (in %)
EQ‐index at baseline	0.34	0.07	< 0.001	10.5	0.32	0.07	< 0.001	8.6
Age	−0.49	0.26	0.062	1.8	−0.02	0.16	0.896	0.0
Female sex	−3.00	4.78	0.531	0.2	0.91	2.94	0.758	0.0
OAC at 12 months after the index stroke								
No OAC								
VKA	20.09	9.08	0.027	2.5	−0.14	5.66	0.981	0.0
NOAC	14.21	6.65	0.033	2.4	9.25	4.12	0.025	2.4
Random effects								
Intraclass correlation coefficient (ICC)	0.04				0.18			
Centers	33				33			
*n*	218				217			
Marginal *R* ^2^/conditional *R* ^2^	0.260/0.286				0.236/0.377			

Abbreviations: NIHSS, National Institute of Health Scale, EQ‐5D = EuroQOL–5 Dimension instrument for measuring quality of life, TIA = transient ischemic attack. OAC = Oral anticoagulation, NOAC = nonvitamin K–dependent antagonists, VKA = Vitamin‐K antagonists.

*Additionally adjusted for education, NIHSS (National Institute of Health Scale) score on admission, modified Rankin scale (mRS) score on admission ≥ 3, index event (transient ischemic attack (= TIA) vs. ischemic Stroke), endovascular treatment, intravenous thrombolysis, comorbidities (congestive heart failure, chronic obstructive pulmonary disease, hypertension, diabetes mellitus, hypercholesterolemia, smoking, prior vascular event, arterial disease, body mass index ≥ 30, renal impairment, serious adverse events (SAE) within 1 year after the index‐stroke (TIA, stroke, major bleeding, myocardial infarction).

### 3.4 Recurrent Vascular Events and Health‐Related Quality of Life

Within 12 months after the index stroke/TIA, 166/3431 (4.8%) patients had a recurrent stroke and 56/3431 (1.6%) patients a TIA. Furthermore, 28/3431 (0.8%) patients had a major bleeding and 35/3431 (1.0%) patients had myocardial infarction. The multivariable linear mixed model showed that none of these types of vascular events were independently associated with lower EQ‐index at 12 months (*p* > 0.05, Table 2). In contrast, EQ‐VAS at 12 months was lower in patients with TIA (mean difference = –6.14, *p* = 0.027), recurrent stroke (mean difference = –4.18, *p* = 0.022), or major bleeding (mean difference = –15.18, *p* = 0.006) (Table 2) but not in patients with myocardial infarction during 12 month follow up (*p* = 0.126).

## 4 Discussion

In this post hoc analysis of the prospective MonDAFIS study, we examined the association of newly detected AF and subsequent OAC on HRQOL in 2495 patients at 12 months after ischemic stroke or TIA. We found that HRQOL at 12 months after the index event depended on AF symptom severity and OAC status. At 12 months, patients with newly detected AF that were anticoagulated had similar HRQOL compared to patients without AF, while patients with newly detected AF that did not receive OAC had lower HRQOL (EQ‐index and EQ‐VAS) than those with anticoagulation. Of note, these effects are independent from stroke severity (assessed by the NIHSS score of the index event), degree of disability or dependence in the daily activities (assessed by the Modified Rankin Scale) and recurrent vascular events. When comparing patients with AF treated with VKA versus NOAC, there was no statistically significant difference in the EQ‐index at 12 months follow‐up. However, another measure of HRQOL, that is, the EQ‐VAS, was statistically significantly lower in patients with AF treated with VKA compared to those treated with NOAC. Patients with symptomatic AF (i.e., an EHRA score ≥ 2) had statistically significantly lower EQ‐index values than patients with asymptomatic AF, while there was no effect on EQ‐VAS at 12 months.

Three recent studies (one prospective randomized multicenter trial and two prospective observational studies) included patients with ischemic stroke or TIA and analyzed the association of AF with HRQOL. In one observational study, Tsalta‐Mladenov and colleagues demonstrated lower HRQOL (measured by the SIS 3.0 questionnaire) in 48 stroke patients with AF at 3 months after the index stroke compared to 102 stroke patients without AF (Haeusler et al. 2021). In another observational study, (Romano et al. 2021). HRQOL was measured with the SIS‐16 and EQ5D‐5D‐5L questionnaire. The authors demonstrated that AF in 516 stroke patients (diagnosed in 14% of the study cohort) was negatively associated with an EQ‐VAS ≥ 90 at 3 months after the index event (Witassek et al. 2019) but not with the EQ‐index (dichotomized as 1 vs. < 1). However, it remained unclear in either study whether AF was detected after the index event or known before the stroke. Sadlanova et al. examined HRQOL in stroke patients using the EQ5D‐5D‐3L questionnaire. The authors reported a modestly but statistically significantly higher HRQOL at 12 months after the index stroke in 39 patients with newly detected AF compared to patients without detected AF in the FIND‐AF randomized study assessed by a multivariable multilevel model (*β* = 0.22, *p* = 0.03, for the EQ‐index). As critically discussed by Sadlonova et al. (2021), however, this effect may be due to the small sample size.

The negative association of AF and HRQOL in unselected patients may be mediated by symptoms directly induced by AF. In three AF‐cohorts (not restricted to patients with ischemic stroke or TIA) higher EHRA scores (i.e. a symptom severity score for AF) were negatively associated with HRQOL (Wynn et al. 2014; Witassek et al. 2019; Freeman et al. 2015). Surprisingly, this association was only detected for the EQ‐index in our study. In contrast, an association of OAC and HRQOL has not been demonstrated in stroke patients with AF so far. Data from a cross‐sectional observational study of 333 patients with AF (including 21% with stroke or TIA) suggests that HRQOL in patients with AF treated with OAC was similar to patients without AF. More specifically, NOAC compared to VKA was slightly more beneficial regarding HRQOL with a difference in utilities between NOACs versus VKAs of a 0.0121 (95% CI: 0.0122 to 0.0128) using the two‐part regression analysis (Gabilondo et al. 2021). While this is in line with the results of our study, our results need to be interpreted with caution as the VKA group was considerably smaller than the NOAC group and the benefit of NOAC versus VKA was only present in EQ‐VAS. A recent systematic review of 16 observational studies (*n* = 18,684 patients) found NOAC treatment to be associated with a greater therapy satisfaction compared to VKA treatment in patients with nonvalvular AF or venous thromboembolism, mainly due to a lower treatment burden (Katerenchuk et al. 2021). It is conceivable that a lower treatment burden may positively affect HRQOL. Contreras Muruaga et al. reported that patients with AF treated VKA within the therapeutic range showed similar therapy satisfaction as patients on NOAC (Contreras Muruaga et al. 2017). Whether or not the observed difference between NOAC and VKA in the EQ‐VAS in the MonDAFIS study was related to time‐in‐therapeutic‐range cannot be answered. Even though the MonDAFIS design precludes any causal inference of OAC on HRQOL, we provide strong association of a clinically relevantly lower HRQOL (mean difference of –16.8 for EQ‐index and –7.5 in EQ‐VAS, respectively) in stroke patients with AF without OAC compared to stroke patients with AF on OAC. Future studies are needed to validate these findings.

Our analysis has strengths and limitations: A strength of this study is the large number of study patients with ischemic stroke or TIA without a history of AF reporting HRQOL within a follow‐up of 12 months. Therefore, we were able to control for a wide range of factors with known association with HRQOL. HRQOL was evaluated according to recommended quality standards for reporting quality of life data in clinical trials (Brundage et al., 2011). Another strengths is that HRQOL was available in 3082/3431 MonDAFIS patients (90%) at baseline. However, there are also some limitations: First, HRQOL before the index event was not available, which is a known predictor for poststroke HRQOL. Furthermore, comprehensive data on potential causes for poor QOL including severe comorbidities that may influence HRQOL (e.g., a cancer) were not collected in the MonDAFIS study. As a life‐expectancy of less than 12 months (before the index event) was an exclusion criterion in the MonDAFIS study, the potential bias in this regard seems to be low. Furthermore, we cannot rule out bias based on indication due to a nonrandom OAC treatment exposure of patients with AF. In addition, it remains unclear why 13% of all MonDAFIS patients with newly diagnosed AF were not anticoagulated at 12 months, as specific reasons (e.g., contraindication, patient preference, or OAC side effects) were not assessed.

However, patients with AF who did not receive OAC at 12 months had similar baseline characteristics compared to anticoagulated patients with AF with no clear evidence of a higher bleeding risk (Table S3).

In addition, the widely used EQ‐5D‐3L (Costa et al. 2021) can be questioned as it features limited generic categories, which may result in a lower sensitivity of subtle changes in HRQOL compared to more specific HRQOL questionnaires (Aliot et al. 2014). However, more comprehensive HRQOL‐questionnaires (like SF‐12 and SF‐36) tend to result in a lower response rate, introducing bias. Furthermore, we did not implement the proxy version of the EQ5D questionnaire in our study, so ED5D data had to be discarded in which the EQ5D questionnaire was not completed unreservedly by the patient. Despite the fact that it is difficult to determine what “minimal clinical important difference” (MCID) can be postulated in terms of HRQOL outcomes, a minimal change in the EQ‐index of 0.08–0.12 was proposed as a MCID in a landmark paper, as this corresponds to a change in the mRS score by 1 point (Kim et al. 2015). Accordingly, estimates of a mean difference of 7% and above in the transformed EQ‐index and the EQ‐VAS would be has to be considered as clinically relevant. Thus, all reported statistically significant results in this study with regard to AF and OAC have to be considered as clinically relevant. A further limitation is that we did not collect data on other complementary patient reported outcomes (such as therapy satisfaction or treatment burden) with regard to the therapy with oral anticoagulants in our study.

## 5 Conclusions

This subanalysis of a large stroke study provides evidence that patients with newly diagnosed AF after acute ischemic stroke or TIA are at risk of having a lower health‐related quality of life. Further evidence of the impact of AF on HRQOL in stroke patients is that AF‐related symptom severity was negatively associated with HRQOL (EQ‐index). Our results indicate that the quality of life in patients with stroke or TIA and AF at 12 months depends on OAC status independently of stroke severity and recurrent vascular events. In addition to OAC being indicated in AF patients with stroke regarding stroke prevention, OAC intake is associated with health‐related quality of life. Further studies are warranted to replicate our findings.

## Author Contributions

**Manuel C. Olma**: Data curation, formal analysis, writing–original draft, visualization, software. **Lena Steindorf‐Sabath**: Writing–review and editing, formal analysis, software. **Serdar Tütüncü**: Data curation, writing–review and editing. **Claudia Kunze**: Data curation, project administration. **Cornelia Fiessler**: Methodology. **Paulus Kirchhof**: Investigation, data curation, methodology, conceptualization. **Joanna Dietzel**: Data curation. **Johannes Schurig**: Data curation. **Patrick Oschmann**: Investigation. **Ludwig Niehaus**: Investigation. **Christian Urbanek**: Investigation. **Götz Thomalla**: Conceptualization, writing–review and editing. **Darius G. Nabavi**: Writing–review and editing, conceptualization. **Joachim Röther**: Conceptualization, writing–review and editing. **Ulrich Laufs**: Conceptualization, writing–review and editing. **Roland Veltkamp**: Writing–review and editing, conceptualization. **Peter U. Heuschmann**: Conceptualization, methodology, writing–review and editing. **Karl Georg Haeusler**: Writing–review and editing, project administration, funding acquisition, supervision, conceptualization. **Matthias Endres**: Conceptualization, funding acquisition, writing–review and editing, resources, project administration, supervision.

## Conflicts of Interest

KGH reports speaker's honoraria, consulting fees or study grants from Abbott, Alexion, Amarin, AstraZeneca, Bayer Healthcare, Sanofi, Boehringer Ingelheim, Daiichi Sankyo, Novartis, Pfizer, Bristol‐Myers Squibb, Biotronik, Medtronic, Portola, Premier Research, W.L. Gore and Associates, SUN Pharma, and Edwards Lifesciences.

PK receives research support for basic, translational, and clinical research projects from European Union, British Heart Foundation, Leducq Foundation, Medical Research Council (UK), and German Centre for Cardiovascular Research, from several drug and device companies active in atrial fibrillation, and has received honoraria from several such companies in the past. PK is listed as inventor on two patents held by University of Birmingham (Atrial Fibrillation Therapy WO 2015140571, Markers for Atrial Fibrillation WO 2016012783).

GT has received speaker's honoraria or consulting fees from Acandis, Bayer Healthcare, Boehringer Ingelheim, Covidien, Bristol‐Myers‐Squibb, Portola, Stryker, and Pfizer.

DGN has received speaker's honoraria and consulting fees from AstraZeneca, Bayer, Boehringer Ingelheim, Bristol‐Myers‐Squibb, Daiichi Sankyo, Novartis, and Pfizer.

JR has received speaker's honoraria and consulting fees from Bayer, Boehringer Ingelheim, Bristol‐Myers‐Squibb, Pfizer, Daiichi Sankyo, Alexion, and Astra Zeneca.

UL reports honoraria/reimbursements for lectures, participation in studies, scientific cooperations (with Saarland University), consulting, travel, support (of colleagues) or support of scientific meetings by Amgen, Bayer, Boehringer‐Ingelheim, Daiichi‐Sankyo, MSD, Sanofi, Servier outside the submitted work. RV reports grants, personal fees and other from Bayer, grants from Boehringer, grants and personal fees from BMS, grants from Daiichi Sankyo, grants from Medtronic, personal fees from Javelin, grants from Biogen, grants and personal fees from Pfizer, personal fees from Abbott, personal fees from Astra Zeneca, other from Novartis, outside the submitted work.

RV is an investigator of Imperial BRC and partially funded by the European Union's Horizon 2020 research and innovation programme under grant agreement No. 754517 (PRESTIGE‐AF).

PUH reports grants from Charité—Universitätsmedizin Berlin during study conduct (within MonDAFIS for biometry; member scientific board) and research grants from German Ministry of Research and Education, German Research Foundation, Bavarian State (ministry for science and the arts) (within STAAB COVID‐19), European Union, Charité—Universitätsmedizin Berlin, Berlin Chamber of Physicians, German Parkinson Society, University Hospital Würzburg, Robert Koch Institute, German Heart Foundation, Federal Joint Committee (G‐BA) within the Innovationsfonds, University Hospital Heidelberg (within RASUNOA‐prime; supported by an unrestricted research grant to the University Hospital Heidelberg from Bayer, BMS, Boehringer‐Ingelheim, Daiichi Sankyo), University Göttingen (within FIND‐AF randomized; supported by an unrestricted research grant to the University Göttingen from Boehringer‐Ingelheim), outside the submitted work.

ME reports grants from Bayer and fees paid to the Charité from Amgen, AstraZeneca, Bayer, Boehringer Ingelheim, Bristol‐Myers‐Squibb, Covidien, Daiichi Sankyo, Glaxo Smith Kline, Novartis, Pfizer, and Sanofi.

PO, CU, LN, CK, ST, CF, MO, LS, JS, and JD report no conflict of interests outside the submitted work.

### Peer Review

The peer review history for this article is available at https://publons.com/publon/10.1002/brb3.70248

## Supporting information



Table S1. Demographic and clinical characteristics of patients at baseline
Table S2. Baseline characteristics of patients with or within diagnosed AF within 12 months
Table S3. Baseline characteristics of patients with diagnosed AF within 12 months and type of oral anticoagulation (VKA‐antagonists or NOAC) at 12 months
Table S4a. Health profiles at baseline and after 12 months (stroke patients)
Table S4b. Health profiles at baseline and after 12 months (TIA patients).
Table S5. Quality of life: EQ‐VAS and EQ‐index 12 months after index event analyzed by a univariate analysis
Table S6. Subgroup analysis/model 1—quality of life: EQ‐index and EQ‐VAS at 12 months in patients with or without symptomatic atrial fibrillation (according EHRA score) diagnosed within 12 months after index event analyzed by a multivariable linear mixed model.*

## Data Availability

Deidentified participant data with corresponding data dictionary of the data underlying the current manuscript will be made available upon reasonable request to the corresponding author Prof. Matthias Endres (matthias.endres@charite.de). Data will be shared to external researchers for scientific noncommercial purposes after approval of the proposal by the MonDAFIS steering board including a signed data access agreement.
