# Supplementary material for: The Role of Atrial Fibrillation and Oral Anticoagulation Status in Health‐Related Quality of Life 12 Months After Ischemic Stroke or TIA
Source: Brain Behav. 2025 Jan 8;15(1):e70248. doi: 10.1002/brb3.70248 (PMC11710889; doi:10.1002/brb3.70248)
Supplement: Supplementary file 1 — Table S1. Demographic and clinical characteristics of patients at baseline Table S2. Baseline characteristics of patients with or within diagnosed AF within 12 months Table S3. Baseline characteristics of patients with diagnosed AF within 12 months and type of oral anticoagulation (VKA‐antagonists or NOAC) at 12 months Table S4a. Health profiles at baseline and after 12 months (stroke patients) Table S4b. Health profiles at baseline and after 12 months (TIA patients). Table S5. Quality of life: EQ‐VAS and EQ‐index 12 months after index event analyzed by a univariate analysis Table S6. Subgroup analysis/model 1—quality of life: EQ‐index and EQ‐VAS at 12 months in patients with or without symptomatic atrial fibrillation (according EHRA score) diagnosed within 12 months after index event analyzed by a multivariable linear mixed model.* [file BRB3-15-e70248-s001.docx]

**Supplement**

Coding of the independent variables included in the main model

The multivariable linear mixed models (main models for EQ-index and EQ-VAS at 12 months) were adjusted for: EQ index at baseline, age, sex (female/male), years of education (≤ 8, 9-10, ≥ 11 years), and National Institutes of Health Stroke Scale (NIHSS, ranging from 0 to 42 points with higher scores indicating more severe neurological deficits) at baseline (NIHSS 0, 1-4, ≥ 5), thrombolysis (yes/no), modified Rankin Scale (mRS) at baseline (<3/ ≥ 3), thrombectomy (yes/no), stroke or TIA as an index event, and the dichotomous cardiovascular risk factors, which were either preexisting or diagnosed at baseline: diabetes mellitus, hypercholesterolemia, arterial hypertension, smoking, vascular disease (coronary heart disease and/or peripheral artery disease), chronic obstructive pulmonary disease, renal insufficiency, heart failure, body mass index (BMI, <30/≥ 30 kg/m^2^), stroke prior to index event. In addition, multivariable linear regression was adjusted first severe adverse event within the 12 months of follow up (TIA, stroke [including subarachnoid hemorrhage, ischemic and hemorrhagic stroke], major bleeding [including intracranial, non-intracerebral bleeding and extracranial bleeding], and myocardial infarction) and study center as a random effect.

| **Table S1. Demographic and clinical characteristics of patients at baseline** | | | | | |
| --- | --- | --- | --- | --- | --- |
|  | **Overall**  **(n=3,431)** | **Patients with EQ-5D**  **(n=3,082)** | **Patient without EQ-5D (n=349)** | **p** |  |
|  |  |  |  |  |  |
| Age (mean (SD)) | 66.2 (12.9) | 66.1 (13.0) | 67.8 (12.1) | **0.014** |  |
| Female sex (n; %) | 1,356/3,431 (39.5) | 1,237/3,082 (40.1) | 119/349 (34.1) | **0.032** |  |
| Education in years |  |  |  | 0.208 |  |
| < 9 (n; %) | 990/3,396 (29.2) | 890/3,065 (29.0) | 100/331 (30.2) |  |  |
| 9-10 (n; %) | 461/3,396 (13.6) | 407/3,065 (13.3) | 54/331 (16.3) |  |  |
| ≥ 11 (n; %) | 1,945/3,396 (57.3) | 1768/3,065 (57.7) | 177/331 (53.5) |  |  |
| NIHSS score on admission (median [IQR]) | 2 [1-4] | 2[1-4] | 3[1-5] | **0.012** |  |
| Modified Rankin scale score on admission ≥ 3 (n; %) | 1,224/3,416 (35,8) | 1072/3,081 (34.8) | 152/335 (45.4) | **<0.001** |  |
| Index stroke (n; %) |  |  |  | 0.082 |  |
| TIA (n; %) | 1,030/3,419 (30.1) | 941/3,076 (30.6) | 89/343 (25.9) |  |  |
| Ischemic Stroke (n; %) | 2,389/3,419 (69.9) | 2,135/3,076 (69.4) | 254/343 (74.1) |  |  |
| Endovascular treatment (n; %) | 98/3,401 (2.9) | 82/3,080 (2.7) | 16/321 (5.0) | **0.033** |  |
| Intravenous thrombolysis (n; %) | 745/3,422 (21.8) | 654/3,082 (21.2) | 91/340 (26.8) | **0.022** |  |
| Comorbidities |  |  |  |  |  |
| Congestive heart failure (n; %) | 97/3,391 (2.9) | 91/3,081 (3.0) | 6/310 (1.9) | 0.374 |  |
| COPD (n; %) | 143/3,390 (4.2) | 128/3,080 (4.2) | 15/310 (4.8) | 0.553 |  |
| Hypertension (n; %) | 2,609/3,393 (76.9) | 2,368/3,081 (76.9) | 241/312 (77.2) | 0.944 |  |
| Diabetes mellitus (n; %) | 882/3,394 (26.0) | 793/3,081 (25.7) | 89/313 (28.4) | 0.310 |  |
| Hypercholesterolemia (n; %) | 1,798/3,392 (53.0) | 1,646/3,081 (53.4) | 152/311 (48.9) | 0.136 |  |
| Smoking (n; %) | 1,673/3,403 (49.2) | 1,505/3,065 (49.1) | 168/338 (49.7) | 0.864 |  |
| Prior vascular event (n; %) | 880/3,388 (26.0) | 784/3,078 (25.5) | 96/310 (31.0) | **0.041** |  |
| Arterial disease* (n; %) | 498/3,390 (14.7) | 434/3,081 (14.1) | 64/309 (20.7) | **0.002** |  |
| Body-mass index ≥ 30 kg/m^2^ (n; %) | 865/3,388 (25.5) | 794/3,059 (26.0) | 71/329 (21.6) | 0.096 |  |
| Renal impairment (n; %) | 262/3391 (7.7) | 240/3,081 (7.8) | 22/310 (7.1) | 0.738 |  |

Abbreviation: NIHSS, National Institute of Health Scale, EQ-5D=EuroQOL–5 Dimension instrument for measuring quality of life, TIA=transient ischemic attack. SD standard deviation, IQR interquartile range. P-values were calculated using exact Fisher Test, t-test for independent samples, or Mann-Whitney-U test. For bivariate statistical comparisons of baseline characteristics, patients with missing data were excluded from performed tests. For further details of baseline characteristics of the MonDAFIS study patients available for intention-to-treat see also (Haeusler et al., 2021).*Arterial disease comprises: coronary heart disease and peripheral arterial disease.

| **Table S2. Baseline characteristics of patients with or within diagnosed AF within 12 months** | | |
| --- | --- | --- |
|  | | |
|  | AF within 12 months (n=261) | No AF within 12 months (n=2,666) |
|  |  |  |
| Age (median [IQR]) | 74 (67-78) | 66 [57-76) |
| Female sex (n; %) | 130 (49.8) | 1013 (38.0) |
| Education in years |  |  |
| < 9 (n; %) | 94 (36.3) | 719 (27.1) |
| 9-10 (n; %) | 29 (11.2) | 363 (13.7) |
| ≥ 11 (n; %) | 136 (52.5) | 1575 (59.3) |
| NIHSS score on admission* |  |  |
| 0 points (n; %) | 20 (7.7) | 316 (11.9) |
| 1-4 (n; %) | 168 (64.9) | 1821 (68.5) |
| ≥ 5 (n; %) | 71 (27.4) | 522 (19.6) |
| Modified Rankin scale score on admission ≥ 3 (n; %) | 102 (39.1) | 905 (33.9) |
| Index stroke |  |  |
| TIA (n; %) | 77 (29.6) | 813 (30.5) |
| Ischemic Stroke (n; %) | 183 (70.4) | 1851 (69.5) |
| Endovascular treatment (n; %) | 16 (6.1) | 68 (2.6) |
| Intravenous thrombolysis (n; %) | 74 (28.4) | 574 (21.5) |
| Comorbidities |  |  |
| Congestive heart failure (n; %) | 8 (3.1) | 68 (2.6) |
| Chronic obstructive pulmonary disease (n; %) | 15 (5.8) | 98 (3.7) |
| Hypertension (n; %) | 222 (85.1) | 2,039 (76.5) |
| Diabetes mellitus (n; %) | 65 (24.9) | 680 (25.5) |
| Hypercholesterolemia (n; %) | 134 (51.3) | 1440 (54.0) |
| Current smoker (n; %) | 97 (37.2) | 1329 (50.1) |
| Prior vascular event (n; %) | 75 (28.8) | 661 (24.8) |
| Arterial disease** (n; %) | 48 (18.5) | 372 (14.0) |
| Body-mass index ≥ 30 (n; %) | 66 (25.6) | 676 (25.6) |
| Renal impairment (n; %) | 35 (13.5) | 175 (6.6) |

Abbreviation: NIHSS, National Institute of Health Scale, TIA=transient ischemic attack. IQR interquartile range. *Arterial disease comprises: coronary heart disease and peripheral arterial disease.

| **Table S3. Baseline characteristics of patients with diagnosed AF within 12 months and type of oral anticoagulation (VKA-antagonists or NOAC) at 12 months** | | | | |
| --- | --- | --- | --- | --- |
|  | | | | |
|  | AF & no oral anticoagulation at 12 months (n=34) | AF & VKA at 12 months (n=26) | AF & NOAC at 12 months (n=201) | P-value |
|  |  |  |  |  |
| Age (median [IQR]) | 74 [61-79] | 72 [64-77] | 75 [68-78] | 0.317 |
| Female sex (n; %) | 18 (52.9) | 12 (46.2) | 100 (49.8) | 0.887 |
| Education in years |  |  |  | 0.620 |
| < 9 (n; %) | 12 (35.3) | 7 (26.9) | 75 (37.7) |  |
| 9-10 (n; %) | 4 (11.8) | 5 (19.2) | 20 (10.1) |  |
| ≥ 11 (n; %) | 18 (52.9) | 14 (53.8) | 104 (52.3) |  |
| NIHSS score on admission* |  |  |  | 0.575 |
| 0 points (n; %) | 1 (2.9) | 2 (7.7) | 17 (8.5) |  |
| 1-4 (n; %) | 26 (76.5) | 15 (57.7) | 127 (63.8) |  |
| ≥ 5 (n; %) | 7 (20.6) | 9 (34.6) | 55 (27.6) |  |
| Modified Rankin scale score on admission ≥ 3 (n; %) | 10 (29.4) | 16 (61.5) | 76 (37.8) | 0.034 |
| Index stroke |  |  |  | 0.256 |
| TIA (n; %) | 14 (41.2) | 6 (23.1) | 57 (28.5) |  |
| Ischemic Stroke (n; %) | 20 (58.8) | 20 (76.9) | 143 (71.5) |  |
| Endovascular treatment (n; %) | 2 (5.9) | 4 (15.4) | 10 (5.0) | 0.111 |
| Intravenous thrombolysis (n; %) | 4 (11.8) | 10 (38.5) | 60 (29.9) | 0.039 |
| Comorbidities |  |  |  |  |
| Congestive heart failure (n; %) | 1 (2.9) | 0 (0.0) | 7 (3.5) | 1.000 |
| Chronic obstructive pulmonary disease (n; %) | 2 (5.9) | 2 (7.7) | 11 (5.5) | 0.803 |
| Hypertension (n; %) | 27 (79.4) | 21 (80.8) | 174 (86.6) | 0.383 |
| Diabetes mellitus (n; %) | 6 (17.6) | 6 (23.1) | 53 (26.4) | 0.597 |
| Hypercholesterolemia (n; %) | 15 (44.1) | 17 (65.4) | 102 (50.7) | 0.264 |
| Current smoker (n; %) | 15 (44.1) | 10 (38.5) | 72 (35.8) | 0.646 |
| Prior vascular event (n; %) | 15 (44.1) | 8 (30.8) | 52 (26.0) | 0.102 |
| Arterial disease** (n; %) | 7 (20.6) | 6 (23.1) | 35 (17.5) | 0.673 |
| Body-mass index ≥ 30 (n; %) | 2 (5.9) | 12 (46.2) | 52 (26.3) | 0.001 |
| Renal impairment (n; %) | 7 (20.6) | 4 (15.4) | 24 (12.0) | 0.338 |

Abbreviation: NIHSS, National Institute of Health Scale, TIA=transient ischemic attack. IQR interquartile range. *Arterial disease comprises: coronary heart disease and peripheral arterial disease. *Arterial disease comprises: coronary heart disease and peripheral arterial disease. P-values were calculated using exact Fisher Test or Kruskal-Wallis-test.

| **Table S4a. Health profiles at baseline and after 12 months (stroke patients)** | | | | | | |
| --- | --- | --- | --- | --- | --- | --- |
| at baseline (n = 2,389) | mobility | self-care | usual activities | pain/ discomfort | anxiety/ depression |  |
| no problem (n; %) | 1,294 (60.4) | 1,554 (72.5) | 1,162 (54.2) | 1,481 (69.1) | 1,567 (73.2) |  |
| some problems (n; %) | 739 (34.5) | 468 (21.8) | 787 (36.7) | 597 (27.9) | 519 (24.2) |  |
| extreme problems (n; %) | 110 (5.1) | 121 (5.6) | 194 (9.1) | 65 (3.0) | 56 (2.6) |  |
|  | | | | | | |
| after 12 months (n = 2,034) | mobility | self-care | usual activities | pain/ discomfort | anxiety/ depression |  |
| no problem (n; %) | 1,243 (65.4) | 1,556 (81.9) | 1,244 (65.5) | 958 (50.4) | 1,312 (69.1) |  |
| some problems (n; %) | 633 (33.3) | 287 (15.1) | 560 (29.5) | 818 (43.1) | 544 (28.6) |  |
| extreme problems (n; %) | 24 (1.3) | 56 (2.9) | 96 (5.1) | 123 (6.5) | 44 (2.3) |  |

| **Table S4b. Health profiles at baseline and after 12 months (TIA patients)** | | | | | | |
| --- | --- | --- | --- | --- | --- | --- |
| at baseline (n = 1,030) | mobility | self-care | usual activities | pain/ discomfort | anxiety/ depression |  |
| no problem (n; %) | 757 (79.8) | 834 (88.8) | 740 (78.0) | 718 (75.8) | 749 (79.0) |  |
| some problems (n; %) | 184 (19.4) | 83 (8.7) | 181 (19.1) | 204 (21.5) | 180 (19.0) |  |
| extreme problems (n; %) | 8 (0.8) | 23 (2.4) | 28 (3.0) | 25 (2.6) | 19 (2.0) |  |
|  | | | | | | |
| after 12 months (n = 892) | mobility | self-care | usual activities | pain/ discomfort | anxiety/ depression |  |
| no problem (n; %) | 632 (74.7) | 775 (91.6) | 642 (75.9) | 462 (54.6) | 591 (69.9) |  |
| some problems (n; %) | 212 (25.1) | 52 (6.1) | 178 (21.0) | 327 (38.7) | 231 (27.3) |  |
| extreme problems (n; %) | 2 (0.2) | 19 (2.2) | 26 (3.1) | 57 (6.7) | 24 (2.8) |  |

| **Table S5. Quality of life: EQ-VAS and EQ-index 12 months after index event analysed by a univariate analysis** | | | | | | |
| --- | --- | --- | --- | --- | --- | --- |
|  | | | | | | |
|  | **EQ-index** | | | **EQ-VAS** | | |
|  | *Mean difference* | *SE* | *p* | *Mean*  *difference* | *SE* | *p* |
|  |  |  |  |  |  |  |
| EQ-index at baseline | 0.47 | 0.02 | <0.01 | 0.36 | 0.02 | <0.01 |
| age | -0.54 | 0.05 | <0.01 | -0.34 | 0.03 | <0.01 |
| Female sex | -7.00 | 1.27 | <0.01 | -1.86 | 0.78 | 0.02 |
| Education in years |  |  |  |  |  |  |
| < 9 |  |  |  |  |  |  |
| 9-10 | 6.77 | 2.07 | <0.01 | 4.26 | 1.27 | <0.01 |
| ≥ 11 | 11.95 | 1.44 | <0.01 | 6.83 | 0.89 | <0.01 |
| NIHSS score on admission* |  |  |  |  |  |  |
| 0 |  |  |  |  |  |  |
| 1-4 | -6.41 | 1.94 | <0.01 | -3.82 | 1.20 | <0.01 |
| ≥ 5 | -18.54 | 2.27 | <0.01 | -9.93 | 1.40 | <0.01 |
| Modified Rankin scale score on admission ≥ 3 | -13.01 | 1.30 | <0.01 | -7.35 | 0.80 | <0.01 |
| Index stroke |  |  |  |  |  |  |
| TIA |  |  |  |  |  |  |
| Ischemic Stroke | -4.76 | 1.35 | <0.01 | -4.26 | 0.82 | <0.01 |
| Endovascular treatment | -5.07 | 3.80 | 0.18 | -2.39 | 2.33 | 0.31 |
| Intravenous thrombolysis | 0.29 | 1.50 | 0.85 | 1.67 | 0.92 | 0.07 |
| Comorbidities |  |  |  |  |  |  |
| Congestive heart failure | -16.84 | 4.09 | <0.01 | -10.45 | 2.51 | <0.01 |
| Chronic obstructive pulmonary disease | -14.63 | 3.31 | <0.01 | -12.08 | 2.02 | <0.01 |
| Hypertension | -11.20 | 1.46 | <0.01 | -7.98 | 0.89 | <0.01 |
| Diabetes mellitus | -10.57 | 1.42 | <0.01 | -5.49 | 0.88 | <0.01 |
| Hypercholesterolemia | -0.57 | 1.25 | 0.65 | -1.18 | 0.77 | 0.12 |
| Current smoker | -0.25 | 1.25 | 0.84 | -0.84 | 0.77 | 0.27 |
| Prior vascular event | -10.92 | 1.44 | <0.01 | -6.12 | 0.89 | <0.01 |
| Arterial disease* | -11.55 | 1.79 | <0.01 | -8.28 | 1.10 | <0.01 |
| Body-mass index ≥ 30 kg/m^2^ | -6.37 | 1.43 | <0.01 | -2.57 | 0.88 | <0.01 |
| Renal impairment | -15.78 | 2.42 | <0.01 | -9.47 | 1.49 | <0.01 |
| SAE within 1 year after the index-stroke |  |  |  |  |  |  |
| TIA | -11.38 | 4.80 | 0.02 | -8.41 | 2.94 | <0.01 |
| Stroke | -13.41 | 3.06 | <0.01 | -8.78 | 1.89 | <0.01 |
| Major bleeding | -15.97 | 9.55 | 0.09 | -23.05 | 5.78 | <0.01 |
| Myocardial infarction | -17.59 | 6.98 | 0.01 | -14.16 | 4.28 | <0.01 |
| Atrial fibrillation | -5.48 | 2.20 | 0.01 | -4.69 | 1.35 | <0.01 |
| Oral anticoagulation at 12 months after the index stroke |  |  |  |  |  |  |
| No anticoagulation |  |  |  |  |  |  |
| OAC | -4.02 | 1.88 | 0.03 | -4.93 | 1.15 | <0.01 |

Abbreviation: NIHSS, National Institute of Health Scale, EQ-5D=EuroQOL–5 Dimension instrument for measuring quality of life, TIA=transient ischemic attack. SAE= serious adverse events. OAC=oral anticoagulation.
*Arterial disease comprises: coronary heart disease and peripheral arterial disease.

| **Table S6: Subgroup analysis / model 1 - Quality of life: EQ-index and EQ-VAS at 12 months in patients with or without symptomatic atrial fibrillation (according EHRA-Score) diagnosed within 12 months after index event analysed by a multivariable linear mixed model*** | | | | | | | | | |
| --- | --- | --- | --- | --- | --- | --- | --- | --- | --- |
|  | **EQ-index**  at 12 months after the index event | | | | **EQ-VAS**  at 12 months after the index event | | | |  |
|  |  | | | |  | | | |  |
| **Fixed effects** | *Mean difference* | *SE* | *p* | *R^2^ (in %)* | *Mean difference* | *SE* | *p* | *R^2^ (in %)* |  |
| EQ-index at baseline | **0.41** | 0.07 | **<0.001** | **15.6** | **0.29** | 0.08 | **0.001** | **6.4** |  |
| age | -0.49 | 0.26 | 0.060 | 2.1 | -0.03 | 0.18 | 0.863 | 0.0 |  |
| Female sex | 1.71 | 4.88 | 0.726 | 0.1 | 1.93 | 3.29 | 0.557 | 0.2 |  |
| EHRA-Score |  |  |  |  |  |  |  |  |  |
| 1 |  |  |  |  |  |  |  |  |  |
| 2 | **-9.04** | 4.44 | **0.042** | **2.4** | -0.66 | 3.04 | 0.829 | 0.0 |  |
| ≥ 3 | **-19.06** | 7.98 | **0.017** | **3.3** | -5.17 | 5.58 | 0.354 | 0.5 |  |
| **Random effects** | | | | | | | | | |
| Intraclass correlation coefficient (ICC) | 0.00 | | | | 0.17 | | | |  |
| Centers | 32 | | | | 32 | | | |  |
| n | 195 | | | | 194 | | | |  |
| Marginal R^2^ / Conditional R^2^ | 0.306 / - | | | | 0.203 / 0.335 | | | |  |

Abbreviation: NIHSS, National Institute of Health Scale, EQ-5D=EuroQOL–5 Dimension instrument for measuring quality of life, TIA=transient ischemic attack. EHRA=European Heart Rhythm Association
*additionally adjusted for education, NIHSS (National Institute of Health Scale) score on admission, modified Rankin scale (mRS) score on admission ≥ 3, index event (transient ischemic attack (=TIA) vs. ischemic Stroke), endovascular treatment, intravenous thrombolysis, oral anticoagulation status at 12 months after the index-stroke, comorbidities (congestive heart failure, chronic obstructive pulmonary disease, Hypertension, diabetes mellitus, Hypercholesterolemia, smoking, prior vascular event, arterial disease, body-mass index ≥ 30, renal impairment, serious adverse events (SAE) within 1 year after the index-stroke (TIA, stroke, major bleeding, myocardial infarction).
